# Trends in lipid profiles and descriptive characteristics of U.S. adults with and without diabetes and cholesterol-lowering medication use—National Health and Nutrition Examination Survey, 2003–2012, United States

**DOI:** 10.1371/journal.pone.0193756

**Published:** 2018-03-06

**Authors:** Carla I. Mercado, Edward Gregg, Cathleen Gillespie, Fleetwood Loustalot

**Affiliations:** 1 Division of Diabetes Translation, National Center for Chronic Disease Prevention and Health Promotion, Centers for Disease Control and Prevention, Atlanta, GA, United States of America; 2 Division for Heart Disease and Stroke Prevention, National Center for Chronic Disease Prevention and Health Promotion, Centers for Disease Control and Prevention, Atlanta, GA, United States of America; Nagoya University, JAPAN

## Abstract

**Background:**

With a cholesterol-lowering focus for diabetic adults and in the age of polypharmacy, it is important to understand how lipid profile levels differ among those with and without diabetes.

**Objective:**

Investigate the means, differences, and trends in lipid profile measures [TC, total cholesterol; LDL-c, low-density lipoprotein; HDL-c, high-density lipoprotein; and TG, triglycerides] among US adults by diabetes status and cholesterol-lowering medication.

**Methods:**

Population number and proportion of adults aged ≥21 years with diabetes and taking cholesterol-lowering medication were estimated using data on 10,384 participants from NHANES 2003–2012. Age-standardized means, trends, and differences in lipid profile measures were estimated by diabetes status and cholesterol medication use. For trends and differences, linear regression analysis were used adjusted for age, gender, and race/ethnicity.

**Results:**

Among diabetic adults, 52% were taking cholesterol-lowering medication compared to the 14% taking cholesterol-lowering medication without diabetes. Although diabetic adults had significantly lower TC and LDL-c levels than non-diabetic adults [% difference (95% confidence interval): TC = -5.2% (-6.8 –-3.5), LDL-c = -8.0% (-10.4 –-5.5)], the percent difference was greater among adults taking cholesterol medication [TC = -8.0% (-10.3 –-5.7); LDL-c = -13.7% (-17.1 –-10.2)] than adults not taking cholesterol medication [TC = -3.5% (-5.2 –-1.6); LDL-c = -4.3% (-7.1 –-1.5)] (interaction p-value: TC = <0.001; LDL-c = <0.001). From 2003–2012, mean TC and HDL-c significantly decreased among diabetic adults taking cholesterol medication [% difference per survey cycle (p-value for linear trend): TC = -2.3% (0.003) and HDL-c = -2.3% (0.033)]. Mean TC, HDL-c, and LDL-c levels did not significantly change from 2003 to 2012 in non-diabetic adults taking cholesterol medication or for adults not taking cholesterol medications.

**Conclusions:**

Diabetic adults were more likely to have lower lipid levels, except for triglyceride levels, than non-diabetic adults with profound differences when considering cholesterol medication use, possibly due to the positive effects from clinical diabetes management.

## Introduction

In 2012, 29.1 million people in the U.S. had diabetes with 1.7 million new diabetes cases among people aged ≥20 years (National Diabetes Statistics Report, 2014). Since people with diabetes have an increased cardiovascular disease (CVD) risk [1, 2], lipid management along with other risk factors is a particular focus in this population. The 2013 guidelines from the American College of Cardiology and the American Heart Association (2013 ACC/AHA) and the 2016 American Diabetes Association Standard of Medical Care in Diabetes (2016 ADA) provided updated guidance on high blood cholesterol treatment recommendations and the eligibility determination for diabetic adults. If the 2013 ACC/AHA guidelines had been in effect from 2005 through 2012, 88% of diabetic adults would have been eligible for cholesterol-lowering medication [3]. Current guidelines (2013 ACC/AHA and 2016 ADA) potentially increased the number who are now eligible for HMG-CoA reductase (statin) therapy [2, 4]. Since eligibility for cholesterol-lowering medication among diabetic adults focuses predominantly on low-density lipoprotein cholesterol (LDL-c) levels; in the age of polypharmacy, it is important to understand how lipid profile levels differ among U.S. adults with and without diabetes, while accounting for cholesterol-lowering medication use.

Lipid levels have consistently been associated with cardiovascular events risk [1, 5–7]. Although LDL-c levels tend not to be higher among diabetic than non-diabetic adults [8], dyslipidemia and lipid levels associated with CVD presents differently among diabetic adults [9, 10]. Triglyceride (TG) levels tend to be greater among diabetic adults and studies have found that TG, as well as non-high-density lipoprotein (non-HDL-c) or total cholesterol (TC)/high-density lipoprotein (HDL-c) ratio, are better coronary heart disease predictors than LDL-c when compared to non-diabetic adults [9, 10]. Although cholesterol-lowering medication use among diabetic adults has demonstrated to lower CVD incidence and all-cause mortality [11], the most effective strategy for managing diabetic dyslipidemia may require a different treatment regimen than for people eligible for treatment without diabetes [12].

A better understanding of how lipid profile levels differ among diabetic adults while accounting for cholesterol-lowering medication use is needed to demonstrate the burden and distribution of diabetic dyslipidemia in the U.S. and to identify potential opportunities for improvement. Since cholesterol management guidelines have historically emphasized the need for medication use among diabetic adults, understanding how lipid profile levels differ may provide insight on differentiating increased CVD risk in this group. The study objectives were to examine the trends and differences in lipid profile levels among U.S. adults by diabetes status and cholesterol-lowering medication use.

## Materials and methods

The National Health and Nutrition Examination Survey (NHANES) assesses the health and nutritional status of the U.S. population and has been previously described in detail [13]. Briefly, NHANES uses a complex multi-stage probability design to select a sample representative of the civilian, non-institutionalized U.S. resident. The survey entails a home interview which collects a variety of information on demographics, socioeconomic, health conditions, and health-related behaviors followed by a physical examination at a mobile exam center (MEC). Physical exams consist of medical, dental, and anthropometric measurements. Participants were randomly assigned to a laboratory session at the MEC to collect blood samples. Only participants assigned to the morning session were asked to fast at least 9 hours prior to their appointment. All the measures of plasma fasting glucose (FG), hemoglobin A1c, and lipid profile levels (TC, LDL-c, HDL-c, non-HDL-c, and TG) were only available for participants with a morning session. LDL-c was calculated in accordance with the Friedewald equation [14] using the measured values of TC, TG, and HDL-c. Since the Friedewald equation is not valid for TG >400 mg/dL, LDL-c was only calculated for TG ≤400 mg/dL. Of the 11,546 participants with a TG measurement, 262 (2%) had TG>400 mg/dL (109 with diabetes and 153 without diabetes). Non-HDL-c was calculated by subtracting HDL-c from TC (TC–HDL-c). The prescription medication questions were collected during the home interview and obtained information on medication used during the past 30 days from the date of visit. Interviewers directly recorded drug names from the medication bottles, when available. Examination response rates for NHANES cycles of 2003 through 2012 ranged from 70–77%. NHANES protocol has been approved by the National Center for Health Statistics Research Ethics Review Board. Data used in this study were de-identified and are publicly available at https://wwwn.cdc.gov/nchs/nhanes/default.aspx.

From NHANES 2003–2012, we included non-pregnant adults aged ≥21 years from the morning fasting sample who fasted 8 to <24 hours at the time of the MEC visit (n = 10,760). Participants were excluded if they were missing data on diabetes status or cholesterol-lowering medication use (n = 27), or laboratory measures (n = 343). The final sample size was 10,390 participants. Fasting morning sample weights were used for all analyses.

Diabetes was defined as: FG ≥ 126 mg/dL, hemoglobin A1c ≥ 6.5% (48 mmol/mol), answering yes to the question “Have you ever been told by a doctor or health professional that you have diabetes or sugar diabetes?”, or reported taking any diabetes medications. Medication use for diabetes was determined for people with diabetes based on: 1) responding “yes” to either question, "Are you now taking insulin?" or "Are you now taking diabetic pills to lower your blood sugar?", or 2) diabetic medications identified in the prescription medication data files. Prescription diabetic medication classes included nonsulfonylureas, sulfonylureas, insulin, thiazolidinediones, meglitinides, alpha-glucosidase inhibitors, combination medications, or other.

Cholesterol-lowering medication use was determined based as 1) responding “yes” to both of the following questions, "To lower your blood cholesterol, have you ever been told by a doctor or other health professional to take prescribed medicine?" and "Are you now following this advice to take prescribed medicine?" or 2) cholesterol-lowering medications identified in the prescription medication data files. Prescription cholesterol-lowering medication classes considered were HMG-CoA reductase inhibitors (statins) and non-statin cholesterol medication (bile acid sequestrants, cholesterol absorption inhibitors, fibric acid derivatives, combination medications, or other).

### Statistical methods

We estimated the prevalence of adults with and without diabetes overall and by subgroups, including: gender, age (21–39, 40–64, and ≥65 years), race/ethnicity (Mexican-American, non-Hispanic white, and non-Hispanic black), body mass index (BMI) [normal (18.5–<25 kg/m^2^), overweight (25–<30 kg/m^2^), and obese (≥30 kg/m^2^)], poverty-to-income ratio (PIR <100, 100–299, 300–499, and ≥500%), education among those aged ≥25 years (<high school diploma, high school diploma, some college, and college degree), any health insurance coverage (yes/no), any routine place to go for health care (yes/no), and taking cholesterol-lowering medication (yes/no). We calculated population estimates using the Current Population Surveys for 2003–2010 and the American Community Survey for 2011–2012, by averaging the population from the 5 NHANES cycles used in this study: 2003–2004 through 2011–2012. We estimated age standardized means of lipid profile and glycemic (FG and hemoglobin A1c) levels between those with diabetes and without as well as within subgroups of cholesterol-lowering medication use by the direct method using the U.S. Census 2010 population (age groups were in 5 year increments starting with age of 20 years: 20–24, 25–29, ect.). We performed linear regression analyses to test for differences in lipid profile levels (dependent variables) between those with and without diabetes (independent variable), by cholesterol-lowering medication use. We also tested for the differences in glycemic levels (dependent variables) between those taking and not taking cholesterol-lowering medication (independent variable), by diabetes status. We tested for interactions between diabetes status and cholesterol-lowering medication use in association with lipid profile and glycemic levels. We tested for significant linear trends in lipid profile and glycemic levels over time within subgroups of diabetes status and cholesterol-lowering medication use. Lipid profile and glycemic levels were log transformed in the regression analyses to meet the criteria of normally distributed standard errors. Results were back transformed and represent the percent difference for one unit increase in the independent variable (depending on the model either diabetes status, cholesterol medication use, or survey cycle years). We adjusted all linear regression models for age (years), gender, and race/ethnicity (Mexican-American, other Hispanic, non-Hispanic white, non-Hispanic black, and other race/multi-racial). For models with lipid profile levels as dependent variables among all adults, we also adjusted for cholesterol medication use. For models with glycemic levels as dependent variables, we made further adjustments for diabetes status in models among all adults and diabetes medication use for models among diabetic adults. We determined statistically significant results for p-values <0.05. We used STATA version 14.0 to perform all analyses, accounting for the complex sampling design, using the morning fasting subsample weights.

## Results

From 2003 through 2012, 12.2% or about 26 million of U.S. adults aged ≥21 years had diabetes (**Table 1**). Compared to non-diabetic adults, diabetic adults were older (90.5% vs 60.7% aged ≥ 40 years), had greater BMI (62.2% vs 31.3% were obese), had lower PIR (62.0% vs 47.5% with PIR <300%), and lower education attainment (55.6% vs 39.7% with high school diploma or less) (p-values <0.001). Among diabetic adults, 52.2% were taking cholesterol-lowering medication than 14.0% of non-diabetic adults (p-value <0.001).

**Table 1:** Characteristics of US adults aged ≥21 years by diabetes status^a^—National Health and Nutrition Examination Survey, 2003–2012.

	All			No Diabetes			Diabetes		
	N = 10390			N = 8620			N = 1770		
	N sample	% (95% CI)	N population (in millions)	N sample	% (95% CI)	N population (in millions)	N sample	% (95% CI)	N population (in millions)
All	10390	100.0 (100.0, 100.0)	210.5	8616	87.8 (86.9, 88.7)	184.8	1774	12.2 (11.3, 13.1)	25.7
Gender									
Men	5113	48.2 (47.3, 49.1)	101.4	4189	47.8 (46.8, 48.7)	88.3	924	51.4 (48.4, 54.4)	13.2
Women	5277	51.8 (50.9, 52.7)	109.0	4427	52.2 (51.3, 53.2)	96.5	850	48.6 (45.6, 51.6)	12.5
Age group (yrs)									
21–40	3252	35.7 (33.9, 37.4)	75.1	3119	39.3 (37.4, 41.2)	72.6	133	9.5 (7.9, 11.5)	2.4
40–64	4441	46.6 (45.1, 48.0)	98.1	3621	46.0 (44.4, 47.6)	85.0	820	50.6 (47.3, 53.9)	13.0
> = 65	2697	17.8 (16.8, 18.8)	37.5	1876	14.7 (13.7, 15.7)	27.2	821	39.9 (36.5, 43.4)	10.2
Race/Ethnicity^b^									
Mexican-American	1773	8.9 (7.3, 10.8)	18.7	1428	8.7 (7.2, 10.5)	16.1	345	10.6 (7.9, 14.0)	2.7
Non-Hispanic White	4960	78.6 (75.8, 81.2)	165.4	4253	79.6 (77.0, 82.0)	147.1	707	71.4 (66.4, 75.9)	18.3
Non-Hispanic Black	2050	12.4 (10.7, 14.4)	26.1	1599	11.7 (10.1, 13.5)	21.6	451	18.1 (14.9, 21.8)	4.6
Body mass index^c^									
Normal	2879	31.0 (29.6, 32.4)	60.7	2639	33.5 (32.1, 34.9)	57.5	240	13.2 (10.9, 15.4)	3.1
Overweight	3495	33.9 (32.6, 35.1)	66.2	3017	35.2 (33.8, 36.6)	60.3	478	24.6 (21.8, 27.3)	5.9
Obese	3720	35.1 (33.7, 36.6)	68.6	2716	31.3 (29.9, 32.8)	53.8	1004	62.2 (58.7, 65.8)	14.9
Poverty-to-income ratio^d^									
<100%	1860	12.7 (11.6, 13.9)	26.7	1503	12.4 (11.3, 13.7)	22.9	357	14.7 (12.4, 17.3)	3.8
100–299%	4108	36.6 (34.5, 38.7)	77.0	3310	35.1 (33.0, 37.3)	64.9	798	47.3 (43.5, 51.1)	12.1
300–499%	1956	25.6 (23.9, 27.4)	53.9	1684	26.1 (24.4, 28.0)	48.2	272	22.0 (18.6, 25.9)	5.6
> = 500%	1695	25.1 (23.1, 27.2)	52.8	1511	26.3 (24.2, 28.5)	48.6	184	16.0 (13.2, 19.4)	4.1
Education (among those aged 25 yrs or older)^e^									
<High school diploma	2717	18.4 (16.9, 20.0)	38.7	2045	17.2 (15.6, 18.9)	31.8	672	26.6 (23.8, 29.6)	6.8
High school diploma	2237	23.4 (22.0, 24.8)	49.3	1790	22.5 (21.1, 24.0)	41.6	447	29.0 (25.9, 32.5)	7.4
Some college	2539	29.0 (27.6, 30.5)	61.0	2140	29.4 (27.8, 31.0)	54.3	399	26.4 (23.3, 29.6)	6.8
> = College degree	2163	29.2 (26.9, 31.6)	61.5	1921	30.9 (28.5, 33.4)	57.1	242	17.9 (15.2, 21.0)	4.6
Health insurance coverage^f^									
Yes	7988	80.5 (79.1, 81.9)	169.4	6512	79.8 (78.2, 81.2)	147.5	1476	85.8 (83.6, 87.8)	22.0
No	2402	19.5 (18.1, 20.9)	41.0	2104	20.2 (18.8, 21.8)	37.3	298	14.2 (12.2, 16.4)	3.6
Routine place to go for healthcare^g^									
Yes	8846	86.2 (85.2, 87.1)	181.4	7197	85.1 (84.0, 86.1)	157.3	1649	94.1 (92.2, 95.5)	24.2
No	1544	13.8 (12.9, 14.8)	29.0	1419	14.9 (13.9, 16.0)	27.5	125	5.9 (4.5, 7.8)	1.5
Taking cholesterol lowering medication^h^									
Yes	2264	18.7 (17.6, 19.8)	39.4	1323	14.0 (13.0, 15.2)	25.9	941	52.2 (49.6, 54.7)	13.4
No	8126	81.3 (80.2, 82.4)	171.1	7293	86.0 (84.8, 87.0)	158.9	833	47.8 (45.3, 50.4)	12.3

P-value <0.05 for all Pearson Chi-square tests, testing difference of each characteristic between adults with and without diabetes.

a Diabetes defined as: fasting glucose ≥126 mg/dL, hemoglobin A1c ≥6.5, responded yes to the question "Other than pregnancy, have you ever been told by a doctor that you have diabetes?", or taking medication for diabetes.

b When stratifying results by race/ethnicity, 1607 participants identified as other Hispanic, non-Hispanic Asian, and other race/multi-racial were not included due to small numbers. However, they are include in all other analyses.

c The body mass index stratification does not include the 296 underweight participants.

d Ratio of family income to poverty as defined by the US Census Bureau. Information available at http://www.census.gov/hhes/www/poverty/methods/definitions.html#ratio of income to poverty. For 771 participants, information on ratio of family income to poverty was missing.

e Of the 734 participants not included in the education stratified estimates, 5 refused to answer the question, 9 did not know the highest level of school completed, and the remainder were 21–24 years old and not included.

f Participants were asked, "Are you covered by health insurance or some other health-care plan?"

g Based on the response to the question, "Is there a place that you usually go when sick or need advice about health?"

h Based on 1) responding “yes” to both of the following questions, "To lower your blood cholesterol, have you ever been told by a doctor or other health professional to take prescribed medicine?" and "Are you now following this advice to take prescribed medicine?" or 2) cholesterol lowering medication was identified in the prescription medication questionnaire.

Among adults taking cholesterol-lowering medication, the age-standardized LDL-c means were lower among diabetic adults (99.6 mg/dL) than non-diabetic adults (118.7 mg/dL) (Wald p-values = 0.0002) (**Table 2**). Similarly, among those not taking cholesterol-lowering medication, diabetic adults had lower age-standardized means of TC and LDL-c (194.9 and 116.9 mg/dL, respectively) than non-diabetic adults (200.6 and 120.7 mg/dL, respectively) (p-values for TC = 0.002 and LDL = 0.021). However, age-standardized mean TG was higher among diabetic adults taking cholesterol-lowering medication (176.8 mg/dL) than those not taking cholesterol-lowering medication or non-diabetic adults (ranging from 119.8–146.0 mg/dL). Although age-standardized mean HDL-c among diabetic adults taking cholesterol-lowering medication (52.2 mg/dL) was not significantly different than non-diabetic adults taking cholesterol-lowering medication (50.7 mg/dL), it was significantly lower in diabetic adults not taking cholesterol-lowering medication (48.8 mg/dL) (p-value = 0.0017). Although non-HDL-c among adults taking cholesterol-lowering medication were not statistically different between adults with diabetes (135.0 mg/dL) and those without (144.1 mg/dL) (p-value = 0.07), the non-HDL-c levels were significantly lower for diabetic adults taking cholesterol-lowering medication (135.0 mg/dL) than diabetic adults not taking medication (146.0 mg/dL) (p-value <0.001).

**Table 2:** Age standardized^a^ means of lipid profile levels and fasting glucose among US adults aged ≥21 years by diabetes^b^ and cholesterol medication use^c^—National Health and Nutrition Examination Survey, 2003–2012.

	Diabetes			No Diabetes		
	All	Taking cholesterol medication	Not taking cholesterol medication	All	Taking cholesterol medication	Not taking cholesterol medication
	Mean (95% CI)	Mean (95% CI)	Mean (95% CI)	Mean (95% CI)	Mean (95% CI)	Mean (95% CI)
Total Cholesterol(mg/dL)^d^^,^^e^	187.0 (183.35,190.57)	187.2 (183.28,191.21)	194.9 (191.32,198.44)	197.0 (195.88,198.22)	194.8 (187.00,202.68)	200.6 (199.31,201.96)
LDL-cholesterol (mg/dL)^d^^,^^e^^,^^f^	108.0 (105.10,110.86)	99.6 (95.47,103.81)	116.9 (113.57,120.15)	117.4 (116.42,118.48)	118.7 (109.52,127.92)	120.7 (119.55,121.87)
Triglyceride (mg/dL)^d^^,^^e^^,^^f^^,^^g^	151.0 (142.53,159.43)	176.8 (162.90,190.78)	146.0 (138.63,153.33)	121.3 (119.36,123.18)	127.3 (120.25,134.32)	119.8 (117.70,121.95)
HDL-Cholesterol (mg/dL)^d^^,^^e^^,^^g^	48.8 (47.27,50.33)	52.2 (51.06,53.34)	48.8 (47.09,50.58)	55.3 (54.86,55.83)	50.7 (49.20,52.18)	56.0 (55.42,56.50)
Non-HDL-cholesterol (mg/dL)^e^	138.2 (134.53, 141.79)	135.0 (130.82, 139.26)	146.0 (142.18, 149.91)	141.7 (140.50, 142.91)	144.1 (135.40, 152.90)	144.7 (143.33, 146.02)
Fasting Glucose (mg/dL)^d^^,^^f^^,^^g^	152.3 (143.98,160.52)	149.4 (141.80,156.97)	154.4 (147.32,161.40)	97.9 (97.57,98.32)	101.0 (100.07,101.85)	97.6 (97.25,98.02)
Hemoglobin A1c (%)^d^^,^^e^^,^^f^^,^^g^	7.2 (6.98,7.45)	7.9 (7.65,8.05)	7.2 (6.92,7.39)	5.4 (5.35,5.38)	5.5 (5.43,5.54)	5.3 (5.34,5.36)

a Means were age standardized to the U.S. Census 2010 population.

b Diabetes defined as: fasting glucose ≥126 mg/dL, hemoglobin A1c ≥6.5, responded yes to the question "Other than pregnancy, have you ever been told by a doctor that you have diabetes?", or taking medication for diabetes.

c Based on 1) responding “yes” to both of the following questions, "To lower your blood cholesterol, have you ever been told by a doctor or other health professional to take prescribed medicine?" and "Are you now following this advice to take prescribed medicine?" or 2) cholesterol lowering medication was identified in the prescription medication questionnaire.

d P-value <0.05 for mean difference test in adults not taking cholesterol medication between those with diabetes and those without.

e P-value <0.05 for mean difference test in adults with diabetes between those taking and not taking cholesterol medication.

f P-value <0.05 for mean difference test in adults taking cholesterol medication between those with diabetes and those without.

g P-value <0.05 for mean difference test in adults without diabetes between those taking and not taking cholesterol medication.

There were significant interactions (p-values range: 0.010–<0.001) between diabetes status and cholesterol-lowering medication use in association with TC, LDL-c, TG, and non-HDL-c levels (**Table 3**). On average, TC, LDL-c, HDL-c, and non-HDL-c levels were lower among diabetic adults than non-diabetic adults [% difference (95% confidence interval) = -5.2% (-6.8, -3.5), -8.0% (-10.4, -5.5), -11.5% (-13.1, -9.9), and -2.7% (-5.0, -0.4), respectively]. However, TG levels on average were greater for diabetic adults than non-diabetic adults [19.5% (14.8, 24.4)]. For adults taking cholesterol-lowering medication than those not, the percent difference between those with diabetes and those without were greater for TC (-8.0% vs -3.5%), LDL (-13.7% vs -4.3%), and non-HDL-c (-7.2% vs 0.2%) and smaller for TG (15.8% vs 23%).

**Table 3:** Percent difference in lipid profiles among those with and without diabetes^a^ and by cholesterol medication use^b^ and percent difference in glycemic levels among those taking and not taking cholesterol medication^b^ by diabetes status^a^—National Health and Nutrition Examination Survey, 2003–2012.

	**Percent difference between those with and without diabetes**^c^						
** **	**All adults**		**Taking cholesterol medication**		**Not taking cholesterol medication**		**P-value (interaction)**^**e**^
** **	**% Difference**^d^ **(95% CI)**	**P-value**	**% Difference**^d^ **(95% CI)**	**P-value**	**% Difference**^d^ **(95% CI)**	**P-value**	
Total Cholesterol	-5.2 (-6.8, -3.5)	<0.001	-8.0 (-10.3, -5.7)	<0.001	-3.5 (-5.2, -1.6)	<0.001	<0.001
LDL-c	-8.0 (-10.4, -5.5)	<0.001	-13.7 (-17.1, -10.2)	<0.001	-4.3 (-7.1, -1.5)	0.004	<0.001
Triglycerides	19.5 (14.8, 24.4)	<0.001	15.8 (10.1, 21.9)	<0.001	23.0 (16.6, 29.6)	<0.001	0.010
HDL-c	-11.5 (-13.1, -9.9)	<0.001	-10.2 (-12.7, -7.6)	<0.001	-12.8 (-14.9, -10.7)	<0.001	0.051
Non-HDL-c	-2.7 (-5.0, -0.4)	0.027	-7.2 (-10.1, -4.2)	<0.001	0.2 (-2.6, 3.1)	0.885	<0.001
	**Percent difference between those taking and not taking cholesterol medication**^**f**^						
** **	**All adults**		**No Diabetes**		**Diabetes**		**P-value (interaction)**^**h**^
** **	**% Difference**^g^ **(95% CI)**	**P-value**	**% Difference**^g^ **(95% CI)**	**P-value**	**% Difference**^g^ **(95% CI)**	**P-value**	
Fasting glucose	1.7 (0.6, 2.8)	0.004	1.8 (1.1, 2.6)	<0.001	0.4 (-4.0, 4.9)	0.870	0.040
Hemoglobin A1c	2.3 (1.6, 3.1)	<0.001	1.9 (1.4, 2.5)	<0.001	0.8 (-1.8, 3.5)	0.550	0.261

a Diabetes defined as: fasting glucose ≥126 mg/dL, hemoglobin A1c ≥6.5, responded yes to the question "Other than pregnancy, have you ever been told by a doctor that you have diabetes?", or taking medication for diabetes.

b Based on 1) responding “yes” to both of the following questions, "To lower your blood cholesterol, have you ever been told by a doctor or other health professional to take prescribed medicine?" and "Are you now following this advice to take prescribed medicine?" or 2) cholesterol lowering medication was identified in the prescription medication questionnaire based on prescription bottles.

c Percent difference [% mg/dL difference in the dependent variable (lipid profile measures) between those without diabetes to those with diabetes] were estimated using linear regression and adjusting for age, gender, and race/ethnicity. For models among all adults, cholesterol medication use was also included in the model.

d Since lipid profile levels were log transformed as dependent variables in the linear regression models, coefficients were back transformed and represent average percent difference in levels between those with and without diabetes.

e P-value testing for interaction between diabetes status and cholesterol lowering medication use in association with lipid profile levels adjusted for age, gender, and race/ethnicity.

f Percent difference in glycemic levels [% difference in the dependent variable (glycemic measures) between those not taking cholesterol medications to those taking cholesterol medication] were estimated using linear regression and adjusting for age, gender, and race/ethnicity. For model among all adults, adjusted for age, gender, race/ethnicity, and diabetes status. For models among those with diabetes, adjusted for age, gender, race/ethnicity, and diabetes medication use.

g Since glycemic levels were log transformed as dependent variables in the linear regression models, coefficients were back transformed and represent average percent difference in glycemic measure between those taking and not taking cholesterol medication.

h P-value testing for interaction between diabetes status and cholesterol lowering medication use in association with glycemic levels adjusted for age, gender, and race/ethnicity.

The interaction between diabetes status and cholesterol-lowering medication use in association with glycemic levels was only significant for FG (interaction p-value = 0.040) (**Table 3**). FG and hemoglobin A1c levels, on average, were higher among adults taking cholesterol-lowering medication than those not taking cholesterol-lowering medication [1.7% (0.6, 2.8) and 2.3% (1.6, 3.1), respectively]. For FG, the percent difference between adults taking cholesterol-lowering medication and those not taking cholesterol-lowering medication was greater among non-diabetic adults than among diabetic adults [1.8% (1.1, 2.6) vs 0.4% (-4.0, 4.9)].

For diabetic adults taking cholesterol-lowering medication, there was an average decline in levels of TC [% difference per survey cycle = -2.3% (-3.7, -0.8)], HDL-c [-2.3% (-4.2, -0.2)], and non-HDL-c [-2.3% (-4.2, -0.3)] from 2003–2012 (**Table 4**). There were no significant changes in mean lipid profile or glycemic levels for diabetic adults not taking cholesterol-lowering medication from 2003–2012. Among non-diabetic adults, average levels of TG declined from 2003 through 2012 for those taking [-5.0% (-7.3, -2.7)] and not taking [-1.9% (-3.1, -0.7)] cholesterol-lowering medication; while hemoglobin A1c increased in those taking [0.4% (<0.1, 0.8)] and not taking [0.5% (0.4, 0.7)] cholesterol-lowering medication.

**Table 4:** Linear trends over time^a^ in lipid profile and glycemic levels among US adults aged ≥21 years by diabetes^b^ and cholesterol medication use^c^—National Health and Nutrition Examination Survey, 2003–2012.

	Diabetes and taking cholesterol medication		Diabetes and not taking cholesterol medication		No Diabetes and taking cholesterol medication		No Diabetes and not taking cholesterol medication	
	% Difference^d^ (95% CI)	P-value	% Difference^d^ (95% CI)	P-value	% Difference^d^ (95% CI)	P-value	% Difference^d^ (95% CI)	P-value
Total Cholesterol	-2.3 (-3.7, -0.8)	0.003	-0.6 (-1.8, 0.5)	0.278	-0.7 (-2.0, 0.6)	0.287	-0.3 (-0.7, 0.2)	0.233
LDL-c	-1.8 (-3.8, 0.2)	0.082	0.0 (-2.0, 2.0)	0.963	-0.3 (-2.6, 2.0)	0.773	0.4 (-0.3, 1.0)	0.254
Triglycerides	-3.4 (-7.1, 0.5)	0.088	-2.8 (-5.8, 0.2)	0.072	-5.0 (-7.3, -2.7)	<0.001	-1.9 (-3.1, -0.7)	0.003
HDL-c	-2.3 (-4.2, -0.2)	0.033	-1.0 (-2.3, 0.3)	0.120	0.8 (-0.4, 2.1)	0.199	-0.5 (-1.2, 0.2)	0.155
Non-HDL-c	-2.3 (-4.2, -0.3)	0.022	-0.6 (-2.2, 1.0)	0.448	-1.3 (-3.0, 0.5)	0.168	0.0 (-0.6, 0.6)	0.912
Fasting Glucose	0.5 (-2.3, 3.3)	0.747	0.0 (-2.0, 2.1)	0.994	-0.2 (-0.7, 0.3)	0.422	0.2 (-0.1, 0.5)	0.225
Hemoglobin A1c	0.4 (-0.9, 1.7)	0.535	1.1 (-0.4, 2.5)	0.148	0.4 (<0.1, 0.8)	0.032	0.5 (0.4, 0.7)	<0.001

a Trends in lipid profiles and fasting glucose were estimated using linear regression and adjusting for age, gender, and race/ethnicity. Models for those with diabetes also adjusted for diabetes medication.

b Diabetes defined as: fasting glucose ≥126 mg/dL, hemoglobin A1c ≥6.5, responded yes to the question "Other than pregnancy, have you ever been told by a doctor that you have diabetes?", or taking medication for diabetes.

c Based on 1) responding “yes” to both of the following questions, "To lower your blood cholesterol, have you ever been told by a doctor or other health professional to take prescribed medicine?" and "Are you now following this advice to take prescribed medicine?" or 2) cholesterol lowering medication was identified in the prescription medication questionnaire.

d Average percent difference in lipid profile and glycemic levels for every two-year survey cycle (2003–2004, 2005–2006, 2007–2008, 2009–2010, and 2011–2012).

## Discussion

One in eight U.S. adults aged ≥21 years had diabetes during 2003–2012. Despite historical cholesterol management guidelines focusing on cholesterol-lowering medication use among people with diabetes, only 52% of diabetic adults were taking cholesterol-lowering medication. However, efforts to better adhere to these guidelines were noted with the increase in cholesterol-lowering medication use among adult with diabetes during 2003 to 2012 [44% in 2003–2004 to 52% in 2011–2012 (p-value = 0.01)], whereas cholesterol-lowering medication use in non-diabetic adults did not significantly change during this time (12% to 16%, p-value = 0.19) (data not shown). Mean TC, LDL-c, and HDL-c levels were significantly lower among diabetic adults than non-diabetic adults regardless of cholesterol-lowering medication use. In fact, a significant interaction was observed between diabetes status and cholesterol-lowering medication use with mean TC, LDL-c, TG, and non-HDL-c levels, implying that U.S. diabetic adults taking cholesterol-lowering medication have better management of their TC, LDL-c, and non-HDL-c levels, but worse control of TG levels, than non-diabetic adults taking cholesterol-lowering medication. Although mean TC, LDL-c, HDL-c, and non-HDL-c levels did not change from 2003 to 2012 for U.S. non-diabetic adults or those not taking cholesterol-lowering medication; there was a significant decline in mean TC levels for U.S. diabetic adults taking cholesterol-lowering medication which could be due to the increase in cholesterol-lowering medication use, medication dosage, or better medication adherence among diabetic adults during this time.

Even though diabetic adults had lower TC, LDL-c, and non-HDL-c levels than non-diabetic adults, they had significantly greater TG and lower HDL-c levels. Furthermore, diabetic adults taking cholesterol-lowering medication had mean TG levels near 177 mg/dL compared with 146 mg/dL among diabetic adults not taking cholesterol-lowering medication. Although this was a cross-sectional study, it is possible that diabetic adults taking cholesterol-lowering medication may have additional comorbidities partially explaining the higher TG levels, such as hypertension, than those not on medication. In this study, 45% of diabetic adults taking cholesterol-lowering medication had hypertension than the 25% of diabetic adults not taking cholesterol-lowering medication, implying that those taking the cholesterol-lowering medication have a greater CVD risk than those not on the medication. Despite the potential increased CVD risk among diabetic adults taking cholesterol-lowering medication due to greater TG levels and more hypertension, those adults did have lower TC and LDL levels than diabetic adults not taking cholesterol-lowering medication. Since studies have found that TG levels as well as non-HDL-c may be better predictors of CVD risk and all-cause mortality than LDL-c among adults with type 2 diabetes [9, 10, 15, 16], consideration of TG management is necessary when treating diabetic dyslipidemia and potentially considering combination therapy rather than statins alone [12, 17, 18] in addition to glycemic control and intensive lifestyle changes.

Although we expected to find mean TC and LDL-c levels to be lower among adults taking cholesterol-lowering medication than those not [% difference (adjusted for age, gender, race/ethnicity, and diabetes): TC = -12.8%, p-value<0.001; LDL-c = -23.0% (p-value<0.001)], we did not expect that among adults not taking cholesterol-lowering medication, the mean TC and LDL-c levels were greater for non-diabetic adults than diabetic adults. We also anticipated lipid profile levels among those taking cholesterol-lowering medication to be similar regardless of diabetes status, not significantly lower among diabetic adults than non-diabetic adults. One reason could be that diabetic adults are more likely to be on a higher intensity medication or diabetic adults may be more adherent to their medications than non-diabetic adults, data not available in NHANES. It is also possible that diabetic adults on average may change their lifestyle, including changes in diet and amount of physical activity, to help manage their condition as a result of national efforts such as the National Diabetes Education Program [19] and therefore have better management of their TC and LDL-c levels.

From 2003–2012, there were no linear differences in mean TC, LDL-c, or HDL-c levels for non-diabetic adults or for those not taking any cholesterol-lowering medication. However, TC, HDL-c, and non-HDL-c levels significantly decreased during this time for diabetic adults taking cholesterol-lowering medication. The declining trends in TC and LDL-c among diabetic adults has been previously documented and believed to be a result of increased uptake of cholesterol-lowering medication use in this population [20], improvements in clinical management of diabetes [21], and an increase in individual diabetes management [22, 23]. Additionally, a decline in diabetes-related complications including myocardial infarction and stroke was noted [24]. Although there were no significant linear changes in TG levels from 2003–2012 among diabetic adults, a decrease was seen for non-diabetic adults with a greater decline for non-diabetic adults taking cholesterol-lowering medication than non-diabetic adults not taking the medication. Regardless of the decline in TC levels among diabetic adults taking cholesterol-lowering medication, the decline in HDL-c and no change in TG levels may suggest a more comprehensive approach that focuses on all lipid profile levels when managing diabetic dyslipidemia.

This study has limitations. First, since information on medication dosage and medication adherence/persistence were not obtained in NHANES, we were not able to control for the confounding effect of dosage and adherence/persistence in our analyses. Therefore, our estimates may be subject to residual confounding especially if there is differential prescription dosage by diabetes status or for new users who have not had sufficient time to demonstrate lipid profiles reflecting the effects of the medication. If diabetic adults have been prescribed higher dosage medication because of their increased risk than non-diabetic adults, this could result in lower lipid values among diabetic adults than non-diabetic adults. Second, since NHANES is a cross-sectional survey, interpretation of the interaction between diabetes status and cholesterol-lowering medication use associated with lipid profile levels should be considered with caution. The differential effectiveness of cholesterol-lowering medication cannot be determined through this study design. Third, another limitation due to the cross-sectional design is that pre-medication lipid profile levels were not available and severity of dyslipidemia when medication began cannot be determined. It is possible that non-diabetic adults may have a more severe condition since treatment eligibility guidelines have had lower cut-points for diabetic adults and controlling or looking into this potential explanation was not possible in this study. Fourth, a potential Age-Period-Cohort effect can be one reason for these findings. The adults with diabetes are an older group compared to adults without diabetes and experiences along with exposures including timing of diabetes onset and changes in treatments may explain some of our findings.

Future studies are needed to better understand the differences in cholesterol management and dyslipidemia association with CVD risk in diabetic adults than non-diabetic adults. It is possible that the etiology between lipid profile levels leading to CVD events may differ for diabetic adults than non-diabetic adults. Additionally, it is possible that diabetic adults may achieve more rapid control of their cholesterol condition than non-diabetic adults due to the many lifestyle modification resources available to better manage their sugar levels. Although current guidelines focus on LDL-c levels in determining cholesterol-lowering medication eligibility, the use of cholesterol-lowering medications, particularly statins, will most likely continue to increase among diabetic adults. The ACC/AHA cholesterol management guidelines emphasize the importance of lifestyle modifications, the foundation for diabetes management that may have contributed to the changes and differences in lipid levels among diabetic adults observed in this study. Expanding the framework from established diabetes management programs to address other chronic conditions, such as dyslipidemia or hypertension, may have the potential to also improve those conditions.
